# NF-*κ*B inhibition impairs the radioresponse of hypoxic EMT-6 tumour cells through downregulation of inducible nitric oxide synthase

**DOI:** 10.1038/sj.bjc.6600678

**Published:** 2003-01-28

**Authors:** M De Ridder, D L Van den Berge, V N Verovski, C Monsaert, N Wauters, G A Storme

**Affiliations:** Oncology Center, Cancer Research Unit, Academic Hospital Free University Brussels (A.Z.-V.U.B.), Laarbeeklaan 101, B 1090, Brussels, Belgium

**Keywords:** NF-*κ*B, radiosensitivity, iNOS, hypoxia, lactacystin

## Abstract

Hypoxic EMT-6 tumour cells displayed a high level of inducible nitric oxide synthase (iNOS) and an increased radiosensitivity after a 16 h exposure to lipopolysaccharide, a known activator of nuclear factor-*κ*B (NF-*κ*B). Both iNOS activation and radioresponse were impaired by the NF-*κ*B inhibitors phenylarsine oxide and lactacystin. Contrasting to other studies, our data show that inhibition of NF-*κ*B may impair the radioresponse of tumour cells through downregulation of iNOS.

Nuclear factor-*κ*B (NF-*κ*B) is part of the early response of mammalian cells to ionizing radiation and triggers cellular defence mechanisms. In line, an aberrant regulation of the NF-*κ*B pathway was found to contribute to the intrinsic hypersensitivity of ataxia telangiectasia fibroblasts to radiation (Jung *et al*, 1995). Other studies have shown that disruption of NF-*κ*B signalling by transfection with the super-repressor I*κ*B*α* (Wang *et al*, 1996; Miyakoshi and Yagi, 2000), by indomethacin (Bradbury *et al*, 2001) or by the proteasome inhibitors MG-132 and PS-341 (Pajonk *et al*, 2000; Russo *et al*, 2001) enhances the radiosensitivity of tumour cells. Based on these findings, NF-*κ*B is currently considered to be a promising molecular target for tumour radiosensitisation.

However, all these studies were performed in aerobic cells and it remains unclear whether such a strategy is applicable in the hypoxic tumour microenvironment, which modifies both radiosensitivity and transcriptional responses. One of these hypoxia-responsive genes appears to be inducible nitric oxide synthase (iNOS), which is frequently overexpressed in solid tumours (Thomsen *et al*, 1995). This enzyme utilises L-arginine to produce nitric oxide (NO), which seems to be carcinogenic and proangiogenic (Ambs *et al*, 1998).

The mechanism by which iNOS is induced in tumour cells is not clarified yet, but the iNOS promoter is known to contain specific binding sites for hypoxia-inducible factor 1 (HIF-1) and for NF-*κ*B (Xie *et al*, 1993; Melillo *et al*, 1995). Our recent study (Van den Berge *et al*, 2001) and other reports (Melillo *et al*, 1996) indicate that hypoxia itself does not activate iNOS, but rather sustains the induction of iNOS by proinflammatory stimuli. Many of them, like lipopolysaccharide (LPS), interleukin-1*β* (IL-1*β*) and tumour necrosis factor *α* (TNF *α*), activate iNOS, through the NF-*κ*B signalling pathway and this mechanism may underlie the constitutive NF-*κ*B activity observed during chronic inflammation and tumorigenesis (Karin *et al*, 2002). Besides this, the tumour microenvironment, which is characterised by transient hypoxia/reoxygenation as a result of poorly organized vasculature and sporadic occlusions of blood vessels, may directly activate NF-*κ*B since this factor is sensitive to oxidative stress (Koong *et al*, 1994). Interestingly, the causative role of iNOS-mediated NO synthesis in hypoxia/reoxygenation injury is well established (Toomey *et al*, 2001) and NO appeared to affect the function of different proteins including NF-*κ*B and HIF-1 by their nitrosylation (Palmer *et al*, 2000; Marshall and Stamler, 2001).

Our laboratory has recently shown that activation of iNOS by proinflammatory cytokines in EMT-6 mammary carcinoma cells results in high output of NO, sufficient to reverse hypoxia-induced radioresistance by analogy to chemical NO donors (Mitchell *et al*, 1993; Verovski *et al*, 1996; Janssens *et al*, 1998,^1999^). Moreover, the potency of cytokines to activate iNOS was drastically increased under hypoxic conditions (Van den Berge *et al*, 2001). We therefore speculated that inhibition of NF-*κ*B might impair rather than enhance the radiosensitivity of hypoxic tumour cells through transcriptional downregulation of iNOS.

To verify this hypothesis, we exposed EMT-6 tumour cells to LPS in 1% oxygen, modelling the hypoxic tumour microenvironment, and examined the activation of NF-*κ*B, the induction of iNOS and the hypoxic cell radiosensitivity. Next, we evaluated the effects of lactacystin and phenylarsine oxide (PAO), which inhibit NF-*κ*B at the level of the proteasome and DNA-binding, respectively.

## Materials and Methods

### Chemicals

Lactacystin was purchased from Alexis Corporation (Laufelfingen, Switzerland). Other chemicals were obtained from Sigma Chemical Co. (St Louis, MO, USA), unless otherwise stated.

### Cell culture

Murine mammary adenocarcinoma EMT-6 cells were kindly provided by Dr Edith Lord (University of Rochester, Cancer Center, New York, USA). Cells (passage 15–40) were cultured in RPMI 1640 medium (Gibco, Paisley, UK) supplemented with 10% bovine calf serum (HyClone Laboratories Inc. Logan, UT, USA) in plastic flasks (Greiner, Frickenhausen, Germany).

### Hypoxic treatment

EMT-6 monolayer cultures grown to early confluence were exposed to lactacystin for 3 h or to PAO for 10 min and washed. Afterwards, LPS was added to activate NF-*κ*B and iNOS under hypoxic conditions. To obtain hypoxia, culture flasks were placed in sealed chambers and subjected to repeated vacuum evacuation and injection of nitrogen/CO_2_-balanced gas containing 1% oxygen. Further processing of cells was done as described below.

### iNOS expression by Western blotting

After a 16 h incubation in hypoxia, cell lysates (from 1×10^5^ cells) were resolved in a 7.5% polyacrylamide–SDS gel and transferred onto HyBond super nitrocellulose membrane (Amersham, Buckinghamshire, UK). The membranes were stained with Ponceau to confirm the equal loading of proteins in the different lanes. After destaining, the blots were incubated for 1 h at 20°C with the primary monoclonal antibody to iNOS (Affiniti Research Products, Exeter, UK) and analysed by an immunoperoxidase-based ECL technique (Pierce, Rockford, IL, USA), according to the manufacturer's protocol.

### Determination of nitrite

After iNOS induction, cultures were reincubated during 6 h in normoxia to accumulate nitrite, an oxidative product of NO. The nitrite level in the medium was determined using the Griess reaction as described previously (Van den Berge *et al*, 2001). These values were normalised to 200 000 cells per well, in a 24-well plate.

### NF-*κ*B activity by EMSA

After a 1 h incubation in hypoxia, the binding activity of NF-*κ*B was analysed in nuclear extracts essentially as described elsewhere (Plaisance *et al*, 1997). Based on literature data on the mouse iNOS promoter (Xie *et al*, 1993), the 34 bp NF-*κ*B consensus primers encoding the sequence 5′-AGG ATG TGC TAG **GGG GAT TTT CC**C TCT CTC TCTG -3′ (NF-*κ*B motif in bold) were synthesised by Invitrogen (Merelbeke, Belgium), annealed and labelled with 32P by the Klenow reaction. In competition assays, a 50-fold excess of unlabelled NF-*κ*B consensus or its mutant (Santa Cruz Biotechnology, CA, USA) was included in binding reactions. In supershift assays, monoclonal antibodies against the p65 (C-20) and p50 (D-17) subunits of NF-*κ*B (Santa Cruz Biotechnology, CA, USA) were used.

### NF-*κ*B expression by Western blotting

Nuclear extracts were resolved in a 10% polyacrylamide–SDS gel and stained with the anti-p65 monoclonal antibody (Santa Cruz Biotechnology, CA, USA). Further blotting and ECL analysis were performed as described above for iNOS.

### Radiosensitivity

After 16 h incubation in hypoxia, cells were collected by trypsinisation and the hypoxic cell radioresponse was estimated as described earlier (Van den Berge *et al*, 2001). Briefly, 0.5×10^6^ cells in 100 *μ*l of medium were placed in conical plastic tubes and pellets were produced by centrifugation at 300 **g** for 5 min. Metabolic oxygen depletion in pellets was induced by a 3 min incubation at 37°C prior to radiation. Cell pellets were irradiated at 37°C at doses 0–20 Gy and cell survival was measured by an 8-day colony formation assay.

### Statistics

All assays were repeated at least three times. Data are expressed as means (symbols) with corresponding standard deviations (bars). The radioresponse of the different experimental groups were compared by one-way ANOVA.

## Results

**Figure 1:**
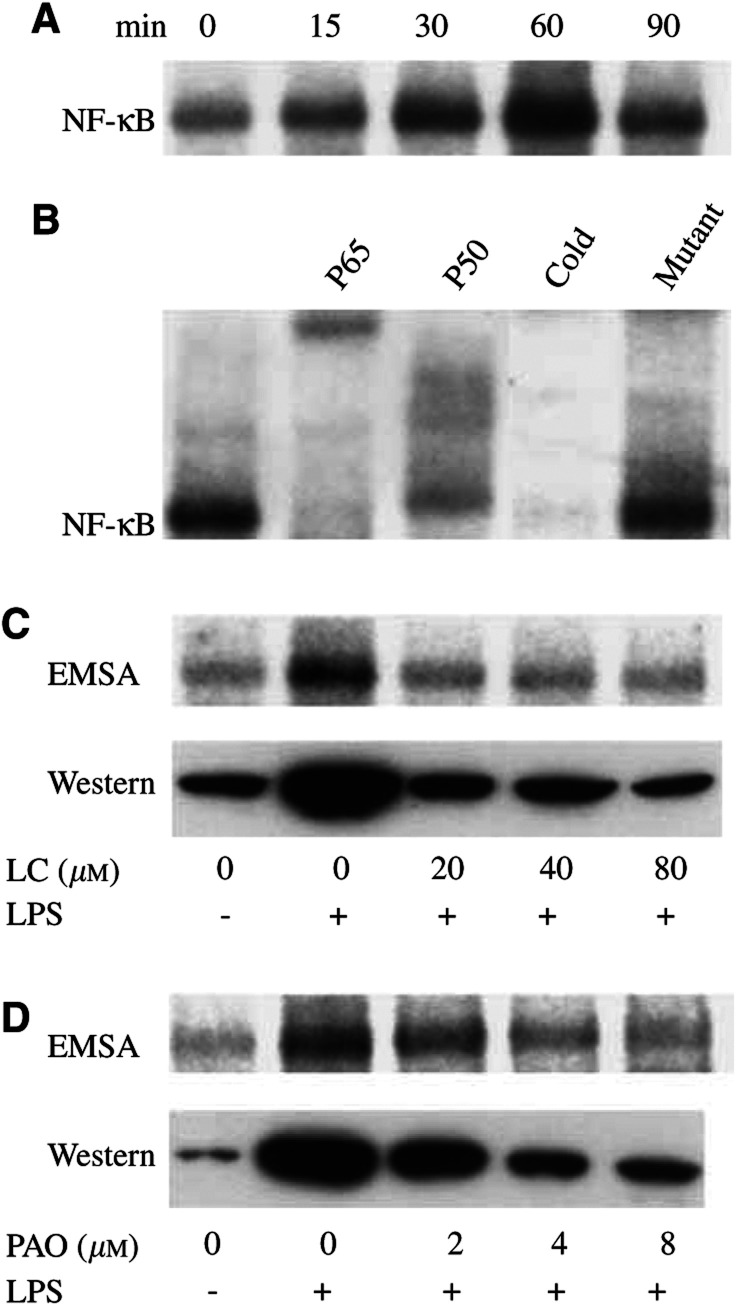
Effect of lactacystin and PAO on nuclear NF-*κ*B expression and binding activity in hypoxic EMT-6 cells. (**A**) Cultures were exposed to 0.1 *μ*g ml^−1^LPS in 1% oxygen for 0–90 min and afterwards analysed for the binding activity of NF-*κ*B in nuclear extracts by EMSA. (**B**) An analysis of the NF-*κ*B composition was performed using anti-p65 and anti-p50 antibodies. The specificity of NF-*κ*B binding was confirmed by inclusion of unlabelled (cold) NF-*κ*B consensus and its mutant in the binding reactions. (**C, D**) The effect of a 3 h pretreatment with lactacystin (LC) and a 10 min pretreatment with PAO on the expression and binding activity of NF-*κ*B was evaluated after a 60 min exposure to 0.1 *μ*g ml^−1^ LPS. The figure is representative of three independent experiments.

EMT-6 cultures were exposed to 0.1 *μ*g ml^−1^ LPS in 1% oxygen to model the reduced oxygenation in solid tumours. Afterwards, the binding activity of NF-*κ*B in nuclear extracts was examined by EMSA. The activation of NF-*κ*B was maximal after a 60 min exposure (Figure 1A

), which was chosen for further experiments. The binding of NF-*κ*B to the probe was blocked by competition with unlabelled NF-*κ*B sequence, but not with its mutant (Figure 1B). The addition of either anti-p65 antibody or anti-p50 antibody induced a supershift, indicating the appearance of the p50/p65 heterodimer in the nuclei. To inhibit NF-*κ*B, cells were pretreated with the proteasome inhibitor lactacystin, which blocks the activation step of NF-*κ*B in the cytoplasm (Musial and Eissa, 2001), or with PAO, which inhibits NF-*κ*B by modifying its vicinal dithiol moiety (Oda *et al*, 2000). Both agents substantially decreased the LPS-induced NF-*κ*B activation as demonstrated in Figure 1C and D. A maximal effect of lactacystin was observed after a 3 h pretreatment at 20–80 *μ*M, while PAO became already active after a 10 min pretreatment at 2–8 *μ*M. In line, Western blots showed a decreased level of nuclear p65, suggesting that the translocation of NF-*κ*B to the nuclei rather than its specific activity was affected.

**Figure 2:**
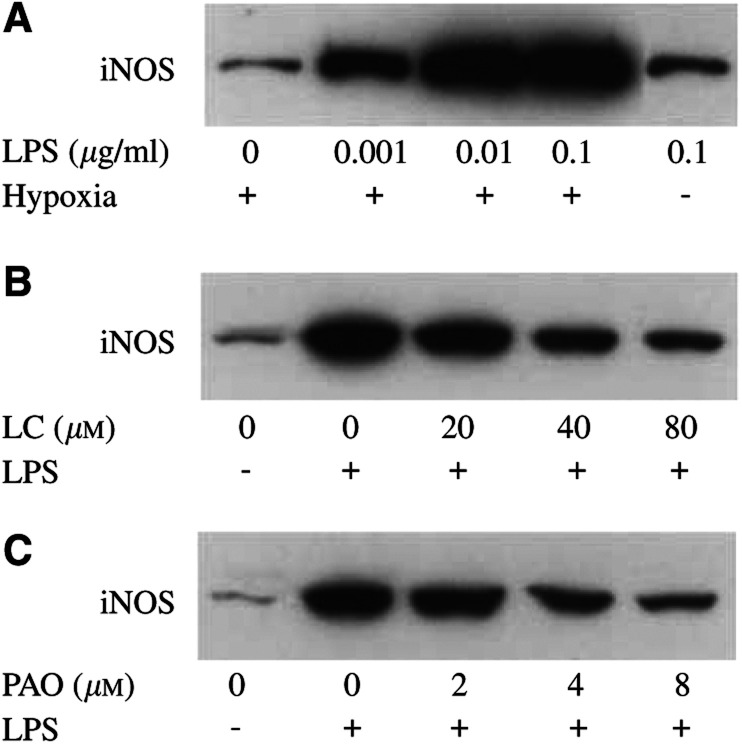
Effect of lactacystin and PAO on iNOS expression in hypoxic EMT-6 cells. (**A**) EMT-6 cultures were exposed to 0.001–0.1 *μ*g ml^−1^ LPS in 1% oxygen and afterwards analysed for the expression of iNOS by Western blotting. The last lane illustrates the iNOS induction by LPS in 21% oxygen. (**B, C**) iNOS expression after a 3 h pretreatment with lactacystin (LC) and a 10 min pretreatment with PAO followed by a 16 h exposure to 0.1 *μ*g ml^−1^ LPS in 1% oxygen. The figure is representative of four independent experiments.

**Figure 3:**
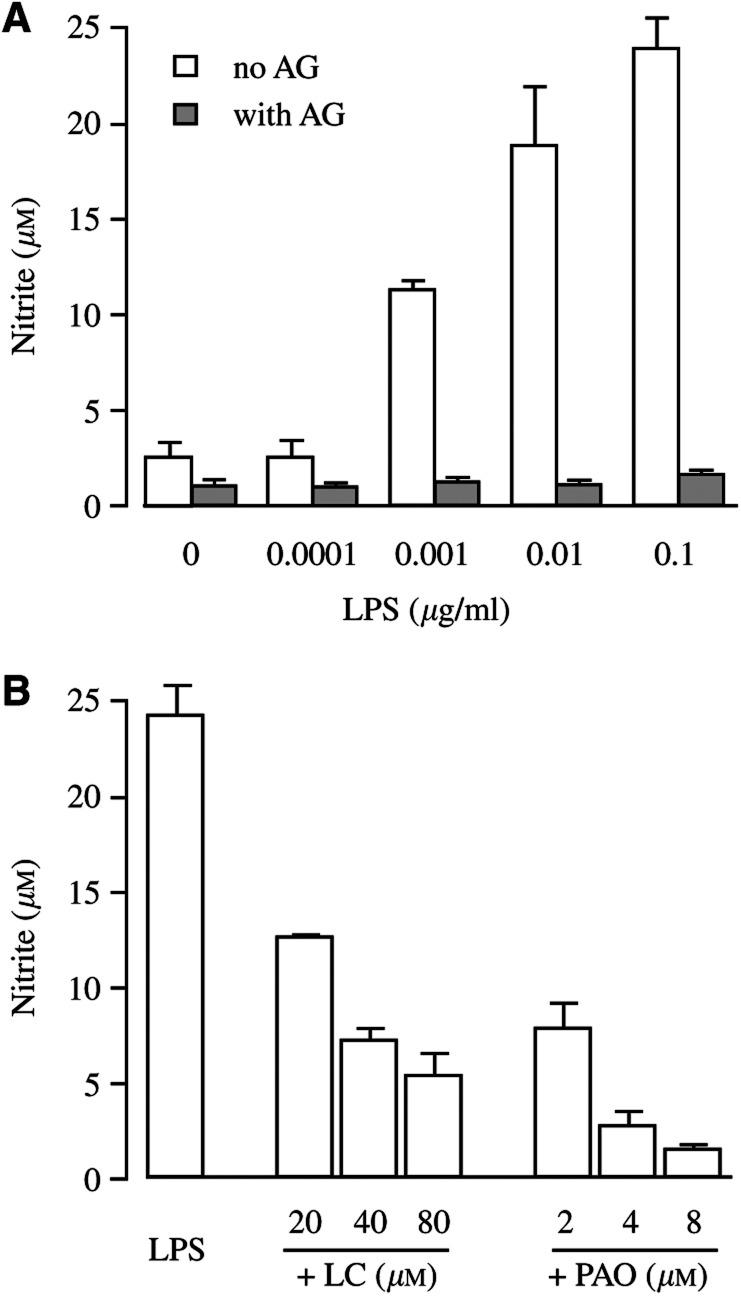
Effect of lactacystin and PAO on nitrite production by EMT-6 cells. (**A**) EMT-6 cultures were exposed to 0.0001–0.1 *μ*g ml^−1^ LPS for 16 h in 1% oxygen and afterwards analysed for the accumulation of nitrite, with or without 3 mM aminoguanidine. (**B**) Nitrite production after a 3 h pretreatment with lactacystin (LC) and a 10 min pretreatment with PAO followed by exposure to 0.1 *μ*g ml^−1^ LPS in 1% oxygen.

To examine whether the NF-*κ*B pathway controls the expression of iNOS, EMT-6 cultures were exposed to 0.001–0.1 *μ*g ml^−1^ LPS for 16 h in 1% oxygen and analysed by Western blotting. LPS induced a dose-dependent activation of iNOS (Figure 2A

), which resulted in accumulation of nitrite, an oxidative product of NO, as determined by the Griess assay (Figure 3A

). The production of nitrite was completely abolished by aminoguanidine, a specific metabolic inhibitor of iNOS. The induction of iNOS by 0.1 *μ*g ml^−1^ LPS was significantly higher in hypoxia compared to normoxia. Both lactacystin and PAO inhibited the expression of iNOS and the production of nitrite at essentially the same concentrations that were efficient to inhibit the activation of NF-*κ*B (Figure 2B, C and 3B). However, both agents–especially at low concentrations–were less potent than aminoguanidine to inhibit the production of nitrite.

**Figure 4:**
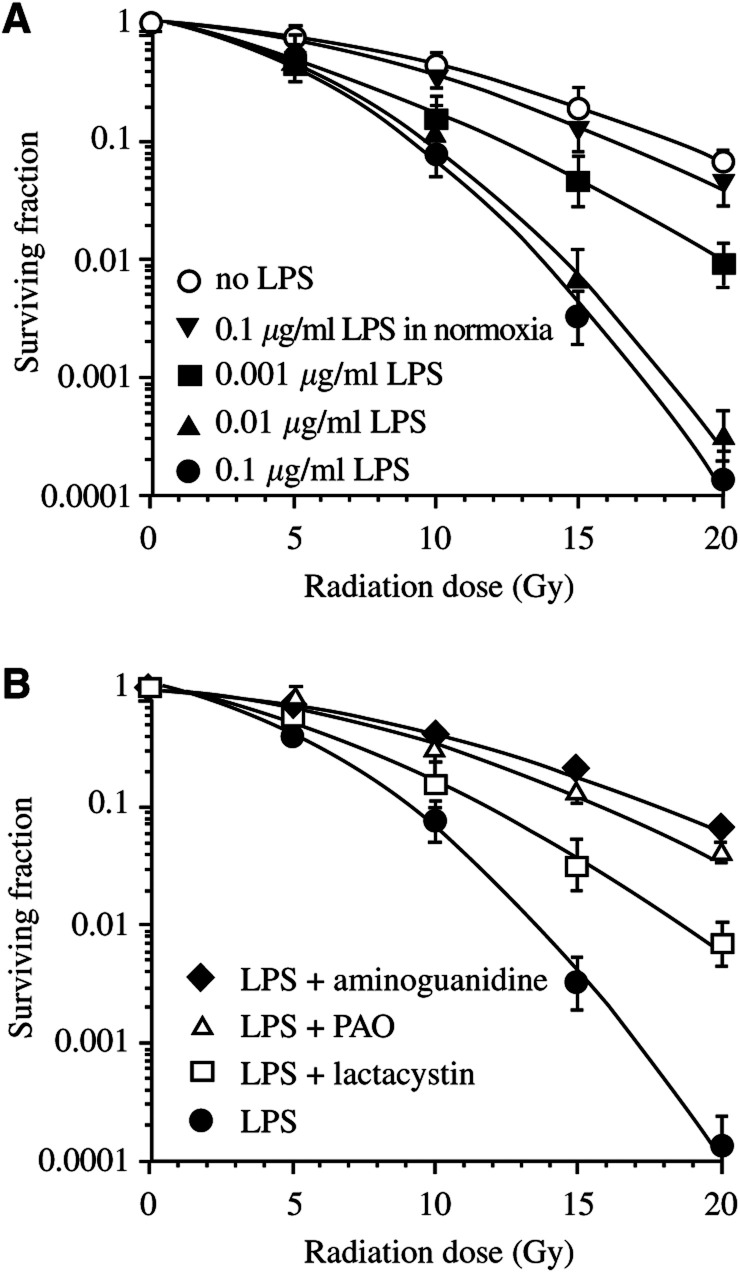
Radioresponse of hypoxic EMT-6 cells after exposure to different concentrations of LPS only (**A**) and pretreated with NF-*κ*B inhibitors followed by LPS treatment (**B**). (**A**) Cells were exposed for 16 h to LPS at 0.001 *μ*g ml^−1^ (▪), 0.01 *μ*g ml^−1^ (▴) and 0.1 *μ*g ml^−1^ (•) in 1% oxygen prior to radiation. The survival curve for nontreated cells (○) and treated with 0.1 *μ*g ml^−1^ LPS in 21% oxygen (▾) are plotted for reference. (**B**) Cells were pretreated for 3 h with 40 *μ*M lactacystin (□) for 10 min with 4 mM PAO (▵) or not pretreated (•) prior to exposure to 0.1 *μ*g ml^−1^ LPS in 1% oxygen. The survival curve for cells treated with 0.1 *μ*g ml^−1^ LPS and 3 mM aminoguanidine is plotted for reference (⧫).

In a final set of experiments, we evaluated the effect of lactacystin and PAO on the hypoxic cellular radioresponse. First, the radiosensitivity of hypoxic EMT-6 tumour cells after a 16 h exposure to 0.001–0.1 *μ*g ml^−1^ LPS in 1% oxygen was examined. We observed a significant increase in hypoxic cell radio-response at all three concentrations with a maximal effect at 0.01–0.1 *μ*g ml^−1^ (*P*<0.001; Figure 4A

). Strikingly, a normoxic incubation with LPS had little, if any, effect on radiosensitivity. To examine whether the radiosensitivity of iNOS-expressing tumour cells may be altered by NF-*κ*B inhibitors, EMT-6 cells were pretreated for 3 h with 40 *μ*M lactacystin or for 10 min with 4 *μ*M PAO and afterwards exposed to 0.1 *μ*g ml^−1^ LPS in hypoxia. Both agents considerably decreased the hypoxic cell radiosensitivity (*P*<0.001; Figure 4B), yet lactacystin was less effective than PAO, in line with Griess data. The iNOS inhibitor aminoguanidine decreased the hypoxic cell radiosensitivity to a level comparable to that of PAO.

## Discussion

Aberrant NF-*κ*B signalling has been associated with several aspects of tumorigenesis, including stimulation of cancer cell proliferation, angiogenesis, formation of metastasis and prevention of apoptosis (Karin *et al*, 2002). Consistently, many tumour cells display constitutively activated NF-*κ*B, which might provide an advantage to survive and to grow in a hypoxic microenvironment. Interestingly, activation of NF-*κ*B is promoted by hypoxia/reoxygenation insults in the vasculature of tumours (Royds *et al*, 1998). Another source of oxidative stress directed to NF-*κ*B are reactive oxygen intermediates produced in a cell type-specific manner by lipoxygenase- and NADPH oxidase-dependent pathways (Bonizzi *et al*, 1999).

NF-*κ*B plays a crucial role not only in tumour development, but also in the response of tumour cells to radiation. Consistently, NF-*κ*B inhibition has repeatedly been used as an approach to radiosensitise tumour cells by stimulating apoptosis or by inhibiting DNA repair (Wang *et al*, 1996; Miyakoshi and Yagi, 2000; Russo *et al*, 2001). We have delineated another downstream target of NF-*κ*B, the cytokine/LPS-inducible form of nitric oxide synthase (iNOS), which may respond to NF-*κ*B inhibitors as well and thereby modulate the radioresponse of tumour cells. Conceivably, this model is relevant for tumours that display constitutive NF-*κ*B signalling to iNOS.

Indeed, the iNOS promoter of mammalian cells encodes specific NF-*κ*B binding sites, which are crucial in the activation of iNOS by inflammatory stimuli (Xie *et al*, 1993). Second, in our model of hypoxic EMT-6 cells, LPS treatment clearly induced translocation of NF-*κ*B to the nucleus and increased its binding activity to the NF-*κ*B consensus of the iNOS promoter, as revealed by Western blotting and EMSA. Third, both the expression and activity of NF-*κ*B and iNOS were inhibited by PAO and lactacystin. This resulted in inhibition of NO production, as determined by accumulation of nitrite, an oxidative product of NO. Finally, PAO and lactacystin significantly decreased the hypoxic radiosensitivity of EMT-6 tumour cells. A similar radioprotection was observed after metabolic inhibition of iNOS by aminoguanidine. These effects may be explained by downregulation of iNOS-mediated synthesis of NO, a potent radiosensitiser that is produced from L-arginine directly in the tumour cells (Janssens *et al*, 1998).

Contrasting to other studies performed in aerobic conditions, our data clearly indicate that NF-*κ*B inhibitors may impair the radiosensitivity of tumour cells and that this effect is unmasked in hypoxia. Therefore, hypoxia-responsive transcription factor(s) are likely to sustain the induction of iNOS by LPS (shown here) or by IL-1*β* and interferon-*γ* (Van den Berge *et al*, 2001). Such a mechanism was first shown in hypoxic macrophages (Melillo *et al*, 1995) and is probably operational in the hypoxic regions of solid tumours wherein iNOS and HIF-1 were found to be active (Thomsen *et al*, 1995; Dachs and Stratford, 1996; Ambs *et al*, 1998). Hence, HIF-1 signalling to iNOS might be another potential target for NF-*κ*B inhibitors, assuming they would be capable of inactivating HIF-1. However, lactacystin is known to increase the level of HIF-1 by blocking its degradation at the level of the proteasome (Salceda and Caro, 1997) and PAO is known to induce HIF-1 by a yet unknown mechanism (Sandau *et al*, 2001). Therefore, the NF-*κ*B pathway is the most likely target to explain the downregulation of iNOS by both inhibitors in EMT-6 tumour cells. In line, lactacystin was shown to inhibit NF-*κ*B by blocking the degradation of its inhibitory unit I*κ*B, thereby inhibiting the induction of iNOS in epithelial HEK293 cells (Musial and Eissa, 2001).

Recently, proteasome inhibitors have emerged as a novel class of radiosensitisers and PS-341, a 26S proteasome inhibitor analogous to lactacystin, has entered multiple phase 2 clinical trials (Elliott and Ross, 2001). This strategy was established *in vitro* using aerobic cultures and therefore addressed the intrinsic radiosensitivity of tumour cells. Till now, no data were available on the radiomodulating effects of NF-*κ*B and proteasome inhibitors in conditions mimicking the hypoxic tumour microenvironment. Our study for the first time demonstrates that NF-*κ*B and proteasome inhibitors may impair the hypoxic radioresponse of iNOS-expressing tumour cells.
